# Modulating streptococcal phenotypes using signal peptide analogues

**DOI:** 10.1098/rsob.220143

**Published:** 2022-08-03

**Authors:** Alec A. Brennan, Mona Mehrani, Yftah Tal-Gan

**Affiliations:** Department of Chemistry, University of Nevada, Reno, 1664 N. Virginia St., Reno, NV 89557, USA

**Keywords:** quorum sensing, bacterial communication, signal peptides, streptococci

## Abstract

Understanding bacterial communication mechanisms is imperative to improve our current understanding of bacterial infectivity and find alternatives to current modes of antibacterial therapeutics. Both Gram-positive and Gram-negative bacteria use quorum sensing (QS) to regulate group behaviours and associated phenotypes in a cell-density-dependent manner. Group behaviours, phenotypic expression and resultant infection and disease can largely be attributed to efficient bacterial communication. Of particular interest are the communication mechanisms of Gram-positive bacteria known as streptococci. This group has demonstrated marked resistance to traditional antibiotic treatment, resulting in increased morbidity and mortality of infected hosts and an ever-increasing burden on the healthcare system. Modulating circuits and mechanisms involved in streptococcal communication has proven to be a promising anti-virulence therapeutic approach that allows managing bacterial phenotypic response but does not affect bacterial viability. Targeting the chemical signals bacteria use for communication is a promising starting point, as manipulation of these signals can dramatically affect resultant bacterial phenotypes, minimizing associated morbidity and mortality. This review will focus on the use of modified peptide signals in modulating the development of proliferative phenotypes in different streptococcal species, specifically regarding how such modification can attenuate bacterial infectivity and aid in developing future alternative therapeutic agents.

## Graphical abstract

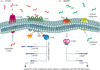

## 1. Streptococci

Bacteria belonging to the genus streptococcus are an incredibly diverse group of organisms with more than 100 different species. Streptococci are nonmotile, non-spore forming, Gram-positive coccoid bacteria, some of which are facultative anaerobes, while others are constitutive anaerobes [1]. Streptococci are members of the endogenous human microflora and greatly influence the immune system. These bacteria are found in almost all organ systems throughout the human body, including the surface of the skin and respiratory tract. They are also among the first bacteria to colonize the oral tract shortly following birth, thereby contributing immensely to dental health and the gut microbiome.

Streptococci use quorum sensing (QS) to control group behaviors and proliferative phenotypes, including competence. The coordinated expression of these responses and phenotypes aids in the continued survival of streptococcal species. QS also aids in advantageous interspecies interactions, promoting microfloral homeostasis and the preservation of a healthy oral microenvironment. In a healthy individual, the proportion of commensal species is far greater than the proportion of pathogenic species. A higher count of commensals is beneficial for improved host immunity against pathogens.

While most streptococci are commensal species, under altered chemical or physiological conditions, many species are opportunistic pathogens. Chemical changes such as altered pH, nutrient content or oxygen concentration, result in an alteration to the physio-chemical environment that may then hinder the growth and survival of certain species while promoting the flourishing of others. Additionally, bacteria can grow in an unregulated fashion in the presence of a weakened or compromised immune system, causing significant overgrowth of certain species and consequent disease.

Previous studies revealed that several different QS circuits play a key role in the enhanced pathogenicity of many streptococci [2,3]. QS allows for effective intraspecies communication, and as a result, bacteria are able to coordinate their responses to promote a successful attack on the host. Such phenotypes can include but are not limited to, competence development, virulence factor production and biofilm formations [4].

## 2. Quorum sensing

It has been understood for decades that both Gram-negative and Gram-positive bacteria are able to communicate and facilitate group responses in a coordinated manner via QS [5]. This method is a ubiquitous cell–cell signalling mechanism that enables bacteria to gauge cell density in a given environment. At a higher cell density, the group then acts in unison to synchronize the transcription of genes associated with group-behaviour phenotypes. This cell-density dependent manner of communication plays a significant role in regulating many bacterial behaviors and phenotypes. QS in Gram-negative bacteria is regulated by the use of small molecules known as *N*-acyl-homoserine lactones (AHLs), whereas Gram-positive bacteria generally utilize autoinducing peptides (AIPs) [3,6]. There is great diversity among these peptide signals—these can be either linear or cyclic and are oftentimes subjected to additional post-translational modifications (table 1). QS is not limited to a singular peptide signal per species but can be regulated by numerous signals. QS-based responses contribute to bacterial survival and proliferation, as well as lend to their overall success in attacking the host and establishing infection.

**Table 1.** Diversity of peptide signalling molecules. Gram-positive species demonstrate a wide variety of peptide signalling molecules.

species	signal	sequence/structure
*Enterococcus faecalis*	GBAP	QN(SPNIFGQWM)^a^
*Staphylococcus aureus*	AIP-I	YST(CDFIM)^a^
*Streptococcus pneumoniae*	CSP1	EMRLSKFFRDFILQRKK
*Streptococcus pneumoniae*	CSP2	EMRISRIILDFLFLRKK
*Streptococcus pyogenes*	SHP2	IMDILIIVGG
		MDILIIVGG
		DILIIVGG—most active
*Streptococcus mutans*	XIP	GLDWWSL
*Streptococcus gallolyticus*	GSP	DFLIVGPFDWLKKNHKPTK

^a^Amino acids in parenthesis represent macrocyclic portions of the peptide.

In Gram-positive bacteria, including streptococci, achieving a bacterial response is reliant upon the primary transcription of a peptide signalling molecule, subsequent post-translational processing to aid with activation and stabilization and finally the secretion of the peptide into the extracellular environment. In general, the peptide signal is secreted and processed by an integral ATP-binding cassette (ABC) transporter. Conversely, the peptide signal can be processed extracellularly, driving the export of the propeptide into the extracellular environment. Once the processed mature peptide reaches a critical concentration threshold, it engages with either a two-component regulatory system or a self-signalling pathway [7,8].

More commonly, the signalling peptide interacts with a two-component signal transduction system (TCSTS) [7]. This pathway is reliant on the combinatory effects of the sensory transmembrane histidine kinase receptor and a corresponding intracellular response regulator. These circuits typically consist of a set of genes with their products labelled Type A-D. The D-type gene product is the propeptide signal that is processed and secreted into the extracellular environment. The B-type gene product is a membrane-bound ABC transporter protein that aids in the processing and secretion of the propeptide signal. The C-type gene product is the first component of the TCSTS, the transmembrane histidine kinase receptor. Lastly, the A-type gene product is the second portion of the TCSTS, or the internal response regulator [6]. TCSTSs are visualized in several Gram-positive species, including *Streptococcus pneumoniae* (Com system), *Staphylococcus aureus* (Agr system) and *Enterococcus faecalis* (Fsr system) [6].

In the TCSTSs, the signalling peptide is initially encoded as a propeptide and is subsequently transcribed, processed and secreted into the extracellular environment. In some systems the processed peptide signal may undergo additional post-translational processing, like cyclization or the removal of *N*- or *C*-terminal amino acids. At a critical threshold concentration, indicative of high cell number, the peptide signal binds to its cognate histidine kinase receptor, lending to the phosphorylation of an internal response regulator, and finally the transcription of both early and late QS-related genes. Early genes are often associated with autoinduction, as the final peptide and protein products help upregulate the transcription of their respective genes. Thus, the mature signalling peptide is commonly referred to as the autoinducing peptide (AIP).

Conversely, the peptide signal can interact with a self-signalling pathway. This is also referred to as the RRNPP circuit, which is named for the response regulators identified from various founding species in this circuit class: **R**ap (*Bacillus subtilis*), **R**gg (*Streptococcus*), **N**prR (*Bacillus cereus*), **P**lcR (*B. cereus*), **P**rgX (*Enterococcus faecalis*) [6,9]. In the self-signalling pathway, the ribosomally synthesized peptide signal is secreted into the extracellular environment. The peptide signal is then processed in the extracellular environment followed by subsequent re-import of the now mature peptide signal. Upon reaching a critical concentration, the mature peptide signal is transported back into the cell by a transmembrane oligopeptide transporter system (Opp) [10]. Once re-imported, the peptide signal complexes with an intracellular receptor/response regulator, promoting dimerization of the regulator and the subsequent upregulation of QS-associated genes.

In either pathway, the TCSTS or RRNPP, interaction of the mature peptide with the transduction system results in the transcription of specific bacterial genes that aid in group behaviour coordination and specific phenotypic response. The majority of Gram-positive bacteria are reliant on AIP-based QS to regulate group behaviours and proliferative phenotypes.

## 3. Competence QS circuits (Com system)

Specific to streptococci, *comX*, also known as *sigX* (*σX*), is an alternative sigma factor that activates the expression of late competence genes. Expression of this gene varies from system to system, however it can be induced by the *comX* inducing peptide (XIP), or the competence stimulating peptide (CSP) [11]. Streptococcal *comX* expression, and thus related proliferative phenotypic response, is directly controlled by one of two different cell-cell signalling systems: ComABCDE (TCSTS) or ComRS (RRNPP), or both. The primary difference between these two systems is the mechanism of pheromone sensing and signal transduction to the *comX* transcriptional activator intracellularly. Thus, both the TCSTS and RRNPP systems play crucial roles in regulating competence in streptococci.

**Figure 1.**
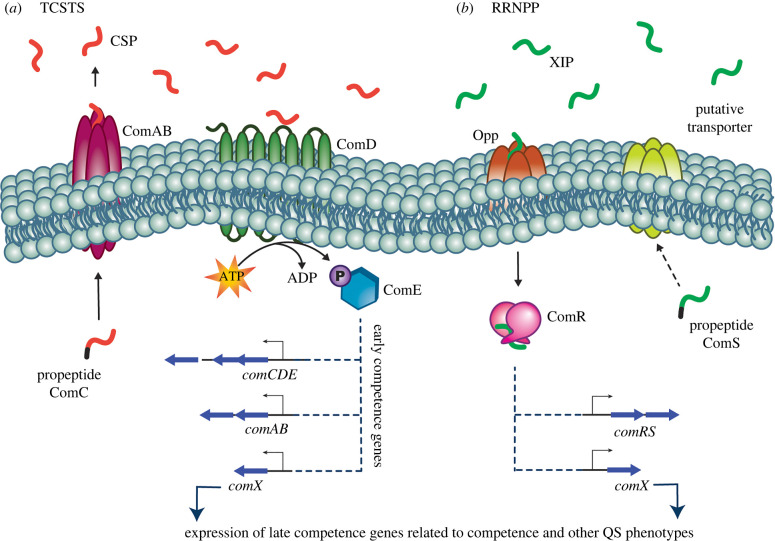
General TCSTS and RRNPP circuits. (*a*) Example of a TCSTS—ComABCDE competence pathway with propeptide ComC, transmembrane histidine kinase receptor ComD and cytoplasmic response regulator ComE. (*b*) Example of the RRNPP circuit—ComRS competence pathway with propeptide ComS and cytoplasmic response regulator ComR. In both systems, the propeptide signal is visualized with the leader sequence that gets cleaved in black, whereas the mature peptide signal is a single colour (red CSP, green XIP).

The well-studied ComABCDE system, prevalent in both the mitis and anginosus groups of streptococci, is a prime example of a prominent TCSTS involved in streptococcal competence regulation (figure 1*a*). This system involves *comC*, which encodes a propeptide processed into its mature form, known as CSP [12]. Once secreted into the extracellular environment, binding of the mature CSP to the transmembrane histidine kinase receptor ComD triggers the phosphorylation and activation of ComE, a cytoplasmic response regulator. Once activated, ComE binds to a conserved promoter sequence, termed p*Ceb*, upstream of QS-associated operons [13]. ComE is then able to facilitate the upregulation of the downstream genes and aid in regulating QS response [11]. Beyond the ComABCDE system, there are several other streptococcal TCSTS and RRNPP pathways aiding in the development of unique phenotypes [7,14].

A second system, ComRS, consists of two genes: *comS* being the precursor peptide to the mature XIP, and a cytosolic receptor encoded by *comR* [15]. Like propeptide ComC, the ComS signal also undergoes post-translational processing, including cleavage from precursor signal sequences, to yield the mature signaling peptide, XIP. An associated oligopeptide permease (Opp) aids in the transportation of the mature peptide from the extracellular environment to the cytosol. Once detected and bound by ComR, the XIP::ComR complex goes on to activate the expression of *comRS* and *comX*, leading to the amplification of the ComRS pathway and lending to the ability of the bacteria to act in unison and control specific phenotypes [16]. It is postulated that there is an associated putative transporter aiding in the maturation and export of ComS, releasing mature XIP into the extracellular environment (figure 1*b*) [17].

## 4. Examples of QS-related phenotypes

### 4.1. Competence

**Figure 2.**
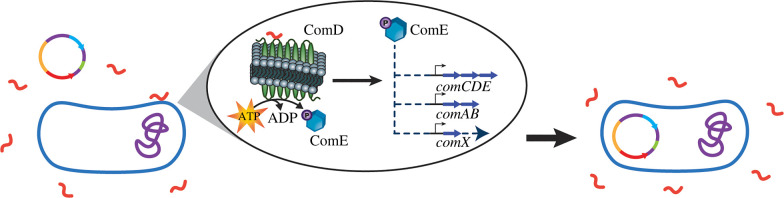
Transformation. Upon exposure to an adequate concentration of peptide signal (CSP in this case), a state of competence is induced in which the bacterium is able to uptake exogenous DNA, including genes for antibiotic resistance, from the surrounding extracellular environment.

Natural genetic competence is the ability of cells to take up exogenous DNA from the environment. This genetic ability has been a potent promoter of genetic variability and subsequent evolution. Competence is accomplished via horizontal gene transfer (HGT) in both Gram-positive and Gram-negative bacteria and has been well conserved in streptococci [18]. Competence for genetic transformation is an induced, temporary state [19]. In streptococci, this state is regulated by the competence regulon. Because this phenotype is so well conserved among streptococci, they maintain an impressive ability to uptake several kilobases of exogenous DNA (figure 2). While this is beneficial for evolutionary purposes, it becomes concerning in a clinical context, as these bacteria can uptake genes for antibiotic resistance and become quite impervious to routine modes of treatment [20].

### 4.2. Biofilm formation

Biofilms are self-assembled exopolysaccharide matrices consisting of one or many bacterial species. Biofilm formation is in part regulated by QS, and as such is a cell-density dependent phenotype [2]. Biofilms aid in protecting the assembled bacterial species, providing some defense against the harsher conditions of the environment they inhabit. This includes protection from antimicrobial agents and antibiotics. While biofilm formation has been evolutionarily selected for, as it is advantageous to bacteria, this phenomenon also contributes to enhanced virulence and pathogenicity [21]. In fact, more than 65% of identified streptococcal infections have shown to be associated with biofilms in some capacity [22].

### 4.3. Virulence

Virulence is defined as the ability of an organism to infect the host and cause disease. Streptococcal species demonstrate a great range of virulence, from those who demonstrate great capacity to cause disease, like *S. pneumoniae* or *S. mutans*, to those that are more often associated with a state of benign commensalism, like *S. oralis*. The process of establishing an infection, including primary colonization, invasion of host cells, systemic toxicity, and evasion of host immune response, is often aided by the production and secretion of virulence factors [23]. These components aid the bacteria in regulating rapid metabolic, morphological and physiological adaptations to overcome host defenses and establish infection [24].

Infection in streptococci can be the result of either direct expansion or vascular invasion [4,24]. Direct expansion occurs when streptococci of a specific niche cause disease in the inhabited area, resulting in disease processes like dental caries, oral abscesses, or pneumonia. Vascular invasion arises following the movement of the bacteria from its inhabited niche into the bloodstream, resulting in infections like sepsis, bacteraemia, meningitis and infective endocarditis.

**Figure 3.**
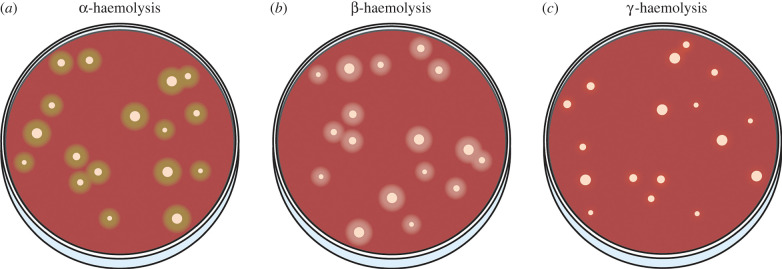
Visualization of haemolysis. (*a*) ɑ-, (*b*) β- and (*c*) γ-haemolytic bacteria.

An example of streptococcal virulence is the induction of haemolysis, or the lysis of red blood cells (RBCs). Streptococci demonstrate all forms of haemolysis, either being ɑ-, β- or γ-haemolytic. ɑ-haemolytic species cause partial lysis of RBCs, resulting in a green hue surrounding bacterial colonies on blood agar media. β-haemolysis is the complete, or true lysis of RBCs and is clearly visualized as a clear zone surrounding colonies. β-haemolysis can be seen in species like *S. pyogenes* or viridans group streptococci [25]. Lastly, γ-haemolysis is the lack of haemolysis, resulting in no change in the blood agar media as the RBCs remain intact (figure 3).

Some streptococcal species use peroxide formation to promote their own growth while preventing the development of neighbouring species [26]. At basal levels peroxide formation can help promote oral health and maintain microfloral homeostasis. However, research has shown that the cytotoxic effect of excess peroxide formation helps bacteria escape from macrophage phagocytosis, thereby promoting efficient colonization and subsequent infection [27]. This can result from a carbohydrate-rich environment, which is often a risk factor for oral disease and a slew of microbial infections.

### 4.4. Interspecies communication

There is a great deal of research detailing the ways in which QS contributes to bacterial pathogenicity, but recent efforts have been put forth to gain insight into the link between QS and bacterial sociality. It has been shown that QS is vital to maintaining homeostasis while promoting coordination between different species. The widespread use of chemical signals not only helps a single species synchronize its own responses but dictates its coordination of behaviors and phenotypes within the larger community [28,29]. This includes contributing to the formation of biofilms, promoting the efficacious exchange of genetic material and maintaining the commensal environment. Unfortunately, disease presents when a given species outcompetes its neighbours and uses QS to its own advantage.

## 5. Present clinical treatment

Presently, bacterial infections are most commonly met with antibiotic treatment for quick resolution. Antibiotics act by inhibiting specific cellular processes in bacteria, commonly by blocking cell wall synthesis or mechanisms linked to cell proliferation, thus resulting in bacteriostatic or bactericidal effects [30]. Antibiotics are classified by their mechanism of action, which can be divided into three main categories: targeting of cell wall synthesis, nucleic acid synthesis or protein synthesis [31].

Bacterial cell walls act as physical, protective barriers and are responsible for maintaining cell integrity. Antibiotics, specifically those belonging to β-lactam and glycopeptide classes, act by preventing the cross-linking of the growing glycan chains and ultimately preventing the growth and stability of the bacterium. Beyond this there are antibiotics that compromise the permeability of the cell membrane, affecting osmotic balance and inducing cell death. Antibiotics that work to disrupt nucleic acid synthesis include quinolones and sulfonamides, among many others [30]. Generally, these target bacterial enzymes that partake in DNA synthesis and replication, as well as limit the presence and availability of folate substrates and cofactors that typically aid in DNA stability and repair. Lastly, there are many antibiotics that work to disrupt protein biosynthesis, including specifically inhibiting bacterial ribosome activity.

**Figure 4.**
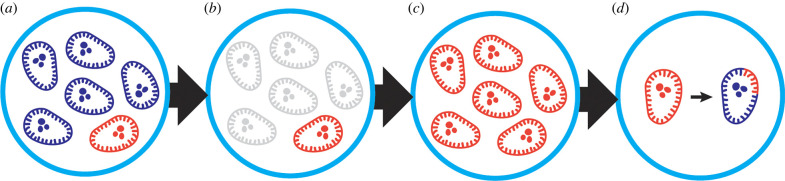
Selection for antibiotic-resistant bacteria. (*a*) Antibiotic treatment begins with the introduction of an antibiotic into an environment with both antibiotic-susceptible (blue) and antibiotic-resistant (red) bacterial species. (*b*) The antibiotic decimates antibiotic-susceptible species, while allowing antibiotic-resistant species to survive. (*c*) This resistant species thrives and proliferates in a now resource-rich environment free of antibiotic-susceptible species. (*d*) Resistant strains maintain the ability to transfer resistance genes to neighbouring species.

While there are many classes of antibiotics that have effectively treated bacterial infections in the past, bacteria have demonstrated rapid development of resistance shortly after the introduction of a new antibiotic, sometimes in less than a single year [32]. Development of resistance can be attributed to the direct pressure imposed by antibiotics. In a neutral state free of antibiotics, antibiotic-resistant strains of bacteria are kept in balance by neighbouring commensals and other potentially antibiotic-susceptible species [33]. However, the introduction of antibiotics leads to an environment in which susceptible species are decimated, leaving behind a nutrient-rich arena where antibiotic-resistant bacteria are now able to thrive and proliferate [31]. This state becomes especially concerning because the now-flourishing antibiotic-resistant strains of bacteria are able to pass these genes for resistance to neighbouring species via HGT. In this case, large populations of bacteria can rapidly gain resistance to a given antibiotic, making established infections and subsequent disease increasingly difficult to treat (figure 4).

The overuse and misuse of antibiotics over the past several decades has led to an increased rate of antibiotic resistance development among bacteria [34]. Streptococci have shown dramatically rising resistance to cephalosporin, beta-lactam and macrolide antibiotic classes, making it substantially more difficult to treat streptococcal infections [35]. To this end, novel methods are needed to treat bacterial infections that avoid the selection for antimicrobial resistance to antibiotics.

## 6. Peptide therapeutics

Peptides have gained a lot of attention in the drug development field and have shown a great deal of potential as viable therapeutic candidates. The initial use of peptides as therapeutic agents began in 1922 upon the introduction of insulin, extracted from animal pancreas, to treat type 1 diabetes [36]. Decades passed with modest use of other peptide agents to treat disease, however, following the advent of solid-phase peptide synthesis (SPPS) in 1963, coupled with purification using high-performance liquid chromatography (HPLC), the field gained significant attention from the pharmaceutical industry [36,37].

There are several advantages of using peptides as therapeutic agents. Peptides are known to have improved selectivity and low toxicity [38]. The selectivity of peptides is essential to many cellular processes and mechanisms, and as such aids in targeting specific receptors and pathways, all while minimizing unwanted interactions with other biological components [37]. This specificity is advantageous when developing peptides aimed to target very particular pathways, such as those associated with hormones. For example, the use of synthetically derived oxytocin will successfully target receptors specific to oxytocin [39]. Whereas synthetic somatostatin will target somatostatin receptors [36,40]. Conversely, peptide-encoding genes for single pathways are commonly well conserved in similar species, allowing for cross-group activity. For example, insulin produced in pigs is a viable candidate for xenotransplantation because its sequence differs by a single amino acid when compared to insulin made in humans [41]. Similarly, genes encoding peptides that target specific pathways are often well conserved across bacterial species. As such, these sequences can be modified and generalized in ways that conserve functionality while establishing effective pan-group modulators, like those used in *S. pneumoniae* QS pathways [36,42,43]. Additionally, peptides have been found to exhibit lower toxicity profiles and decreased immunogenicity compared to other biological probes, like small molecules, further demonstrating their strong potential to serve as future antimicrobial therapeutics [44].

Unfortunately, the use of peptides has also known limitations, including low oral bioavailability and low plasma stability [36,37]. To address these problems, scientists have worked on modifying amino acids and the overall peptide structure to yield more stable and efficient analogues. To prevent proteolysis and other enzymatic degradation of peptides, systematic chemical alterations, including the incorporation of terminal modifications, *N*-methylated residues, D-amino acids or non-proteinogenic amino acids (NPAA) can be used [45]. The addition of *N*- or *C*-terminal modifications aids in preventing enzymatic recognition of the peptide sequence, resulting in improved metabolic stability. Evolutionarily, most mammalian enzymes have developed improved recognition of L-amino acids, making those with D-configuration much more resistant to degradation [36,46]. *N*-Methylation involves the addition of methyl groups in place of amide protons, aiding in protease resistance, as well as membrane permeability, oral bioavailability and enhanced biological activity [47]. NPAAs are not naturally encoded in the genetic code or found in polypeptide chains. The incorporation of NPAA also inhibits peptide proteolysis by stabilizing backbone conformation and/or removing the enzyme recognition site [45]. Lastly, peptide cyclization is also very useful in improving *in vivo* peptide stability [48]. Cyclization reduces conformational freedom, thereby enhancing metabolic stability, binding affinity and specificity to target molecules, as well as improves membrane permeability [49]. Overall, cyclization with natural or NPAAs can improve stability and protease resistance and stabilize the desired conformation of synthetic peptide analogues. These are only a few of the many ways in which peptides can be modified to improve their overall effectiveness and efficiency in serving as potent biological therapeutics.

In recent years more research has been dedicated to designing and utilizing peptide-based probes to understand and attenuate various bacterial mechanisms, especially those related to pathogenesis and virulence. This push has been largely influenced by the ever-increasing prominence of antibiotic-resistant strains of bacteria. As mentioned previously, streptococci have been of particular concern, as this group has demonstrated drastically rising resistance to cephalosporin, beta-lactam and macrolide antibiotics, rendering streptococcal infections increasingly difficult to treat [35]. It has proven advantageous to target the ways in which these bacteria communicate, thereby allowing for the modulation of resultant group behaviors and phenotypes. In Gram-positive bacteria, effective bacterial communication generally relies on the production of a signalling peptide, or AIP. This chemical signal gives bacteria the power to act in unison and coordinate a variety of group behaviors that result in enhanced infectivity, symbiosis or acquirement of antibiotic resistance. Ultimately, in many cases these group behaviors help the bacteria coordinate an effective attack on the host. As such, modification of the peptide signal allows researchers to probe QS communication pathways and define specific bacterial mechanisms. Consequently, modification of the peptide signal also allows for the modulation of resultant group behaviours and phenotypes associated with QS.

The influence of QS on bacterial pathogenicity and virulence, coupled with the rising prevalence of antibiotic resistance among streptococci, has led to a growing interest in targeting QS as an alternative therapeutic approach [3]. The use of peptides is both non-lethal and non-selective, meaning that peptides circumvent the selection for antibiotic resistance among bacterial populations, making it a viable alternative to traditional antibiotic treatment. Several peptide analogues have been developed to serve as QS modulators, meaning they either inhibit or elicit a specific group behaviour or phenotype associated with QS circuits. In doing so, phenotypic expression associated with pathogenicity, like biofilm formation or virulence factor production, can be inhibited and used to attenuate subsequent infection. Conversely, beneficial phenotypes with antimicrobial properties, like hydrogen peroxide formation in *S. sanguinis*, can be elicited to inhibit the growth of nearby pathogenic species [26]. The following section will detail the use of peptide-based probes in modulating the development of proliferative phenotypes in specific streptococcal species.

## 7. Potential application in controlling streptococcal phenotypes

### 7.1. Mitis group: *S. pneumoniae*

*Streptococcus pneumoniae* is an opportunistic human pathobiont that colonizes the nasopharynx of humans and causes a wide variety of severe diseases such as pneumonia, meningitis and bacteraemia [50,51]. Pathogenic pneumococcus alone is responsible for approximately 22 000 deaths and 445 000 hospitalizations in the United States, with an annual healthcare cost of $3.5 billion [52].

QS mechanisms and associated phenotypes in *S. pneumoniae* have been extensively studied [51]. Competence is one of the most well-characterized features of *S. pneumoniae* and is governed by the ComABCDE circuitry [53]. Past studies have revealed a correlation between competence and virulence in *S. pneumoniae*. This relationship was not well understood; however, Guiral and co-workers suggested a mechanism to address this—a process known as allolysis. Here, competent cells can trigger the lysis of non-competent cells, leading to the release of virulence factors, like the cytolytic toxin pneumolysin (Ply) from non-competent cells [18,54,55].

A slew of other factors are also involved in the killing of the non-competent cells, such as LytA, LytC and CbpD, along with a two-peptide bacteriocin (CibAB) [54]. A number of surface components play a vital role in pathogenesis development of *S. pneumoniae* including polysaccharide capsule, *S. pneumoniae*'s cell wall components, Ply, pneumococcal surface proteins, choline-binding proteins (CBPs), lipoproteins, autolysin, LPXTG cell wall-bound proteins, Immunoglobulin A1 (IgA1) protease and pathogenicity islands (PAIs) [56–58]. As such, it is useful to target the production of these components and prevent pathogenesis to begin with.

Biofilm formation is another significant phenotype regulated in part by the competence regulon in *S. pneumoniae* [51,59]. Pneumococcal biofilms are intrinsically antibiotic resistant, and the biofilm matrix facilitates evasion of host immune responses, enabling the persistence and spread of bacteria. Additionally, the extracellular matrix of polysaccharides provides protection and can boost *S. pneumoniae* virulence. According to a recent study by Aggarwal, *briC*, a novel competence gene, encodes BriC and is upregulated by activated ComE [59]. BriC acts as a link between the competence pathway and biofilm production. Inhibition of BriC may help attenuate biofilm formation and associated infection; however, more research is needed to confirm this [28,59].

**Figure 5.**
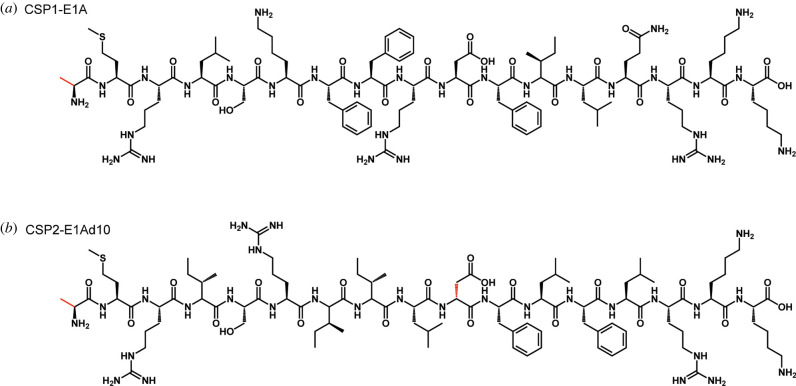
Modified *S. pneumoniae* CSP1 and CSP2 analogues. Structures of the modified CSP sequences, showing (*a*) replacement of glutamate at position 1 with alanine in CSP1 (CSP1-E1A), as well as (*b*) the same modification in CSP2 coupled with a change from L-aspartate to D-aspartate (CSP2-E1Ad10).

In the *S. pneumoniae* competence regulon, multiple CSP and ComD variants are present. Two of the most important specificity groups are group 1 and group 2 that produce two distinct CSP pherotypes: CSP1 and CSP2, along with their cognate receptors: ComD1 and ComD2, respectively [59–61]. In 2012, Duan and co-workers set out to investigate the CSP structures and reveal critical chemical motifs involved in ComD activation through structure-activity relationship (SAR) analysis. It was found that a modified CSP1 analogue with alanine in place of glutamate in position 1 (CSP1-E1A) is capable of competitively inhibiting ComD1 activation (figure 5*a*) [62]. Later, Yang and co-workers have performed a more in-depth analysis of the CSP1 and CSP2 scaffolds. The authors quantified the inhibitory activity of CSP1-E1A and found that it is an effective ComD1 inhibitor with an IC_50_ value of 85.7 nM (table 2). In the same study, the authors found that a double mutant of CSP2, CSP2-E1Ad10, was a powerful ComD2 inhibitor (IC_50_ = 56.5 nM; figure 5*b*). Unfortunately, neither peptide exhibited effective cross-group reactivity (i.e. CSP1-E1A against ComD2 or CSP2-E1Ad10 against ComD1) [61].

**Table 2.** *S. pneumoniae* CSP analogues. Modifications to the native peptide sequences are bolded, with parenthesis indicating cyclization at the noted residues. EC_50_ and IC_50_* values represent the CSP concentration required to achieve 50% of the maximal activation or inhibition of the associated regulon.

peptide name	peptide sequence	EC_50_/IC_50_* (nM) ComD1	EC_50_/IC_50_* (nM) ComD2	references
CSP1	EMRLSKFFRDFILQRKK	10.3	526	[61]
CSP1-E1A	**A**MRLSKFFRDFILQRKK	85.7*	Inactive	[61]
CSP2	EMRISRIILDFLFLRKK	1650	50.7	[61]
CSP2-E1A	**A**MRISRIILDFLFLRKK	>1000*	>1000*	[61]
CSP2-E1Ad10	**A**MRISRIIL**d**FLFLRKK	>1000*	56.5*	[61]
CSP1-E1A-cyc(Dap6E10)	**A**MRLS**(Dap**FFR**E)**FILQRKK	75.8*	182*	[43]

**Figure 6.**
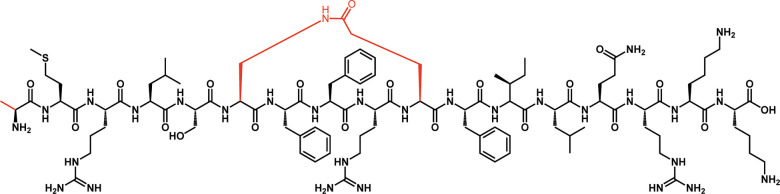
*Streptococcus pneumoniae* CSP-based pan-group QS inhibitor. This modified peptide analogue, termed CSP1-E1A-cyc(Dap6E10), contains two modifications: alanine at position 1 in place of aspartate, as well as a lactam bridge between amino acids 6 and 10.

Due to weak stability against enzymatic degradation and minimal cross-group inhibition, both CSP1-E1A and CSP2-E1Ad10 exhibited limited therapeutic potential. To improve the stability of the peptides and produce pan-group QS modulators, Yang and co-workers have focused on the insertion of a lactam bridge between the sixth and tenth amino acids in the native CSP1 sequence. Following the identification of cyclic pan-group activators, incorporation of the E1A modification has led to the development of a pan-group inhibitor, CSP1-E1A-cyc(Dap6E10) (figure 6) [43]. This peptide demonstrated inhibition of both ComD1 and ComD2, with IC_50_ values of 75.8 nM and 182 nM, respectively (table 2), thereby effectively inhibiting QS.

While the previously discussed peptide analogues demonstrated marked activity as potent inhibitors in the laboratory setting, researchers sought to further elucidate the effects of these peptides on resultant phenotypes *in vivo*, ultimately delineating their effects in modulating infection and disease. Zhu *et al.* demonstrated that the previously identified CSP1-E1A inhibitor peptide also serves as a robust inhibitor of ComD1 in an animal model [63]. In a separate study, mice were intranasally infected with group 2 pneumococcus (TIGR4 strain) and treated with CSP2-E1Ad10 2 h later. Mice exhibited a greater survival rate following CSP2-E1Ad10 treatment compared with the negative control vehicle (PBS), highlighting the potential of CSP2-E1Ad10 in reducing pneumococcal infections caused by group 2 pneumococcus [64].

Another study was conducted to assess the cross-group inhibitory effect of the CSP1-E1A and CSP2-E1Ad10 analogues. D39-infected mice (group 1) were treated with CSP2-E1Ad10 (group 2 inhibitor) and TIGR4-infected mice (group 2) were treated with CSP1-E1A (group 1 inhibitor) [43]. Mice exhibited a 100% fatality rate, indicating that neither inhibitor was effective in preventing cross-group infection. In the same study, the authors also tested the lead inhibitor analogue, CSP1-E1A-cyc(Dap6E10), on mice infected with either group 1 or group 2 pneumococcus. This peptide proved to have pan-group inhibitory activity, as it was able to attenuate pneumococcal infection caused by either D39 or TIGR4, resulting in enhanced survival rates in mice [43]. These encouraging findings highlight the therapeutic potential of peptide analogues, especially the pan-group inhibitor CSP1-E1A-cyc(Dap6E10), to serve as novel therapeutic leads and effective pharmacological agents [58,65].

### 7.2. Bovis group: *S. gallolyticus* subs. gallolyticus

Like many other streptococci, *S. gallolyticus subs. gallolyticus* (*Sgg*), previously known as *S. bovis* biotype I, is a member of the human microbiota and acts as an opportunistic pathogen. This species has been found responsible for causing infective endocarditis and bacteraemia, as well as shown to have a strong correlation with the development of colorectal cancer (CRC) [66]. *Sgg* has shown marked activity in promoting colon cancer cell proliferation and tumor growth, suggesting it may be a bacterial driver of such disease. This bacterium has demonstrated the strongest association with CRC. In fact, it has demonstrated a 7-fold higher risk compared to infections caused by neighbouring species, suggesting that this enhanced virulence may be the result of an *Sgg*-specific pathway [67]. Moreover, a separate study indicated that 68% of patients with CRC are seropositive for *Sgg*-specific IgG antibodies, compared to 17% in a sex- and age-matched control population [66]. Researchers have begun detailed investigations of this species in hopes of elucidating associated virulence mechanisms and methodologies to attenuate infectivity using peptide inhibitors.

In 2018, Harrington and co-workers sought to gain novel insight about the ComABCDE competence regulon QS circuitry and its regulatory role in *Sgg*. Multiple sequence alignments (MSA) of *comC*, a well-preserved gene coding for the CSP signal among streptococcal species, revealed a potential 24-mer CSP signal [68]. Comparison of this signal with those of other streptococci indicated atypical processing of the CSP. Following the cleavage of the near-ubiquitous double-glycine motif preceding the mature CSP, *Sgg* was predicted to produce and secrete a 24-mer CSP. However, because many streptococcal CSPs begin with a negatively charged residue at the *N*-terminus, it was hypothesized that the mature signal was in fact a 21-mer. Extraction of this native peptide signal from bacterial supernatant confirmed that *Sgg* does in fact produce a 21-mer signal, while MS/MS analysis confirmed the previously predicted amino acid sequence [69]. Researchers then sought to define the regulatory role of this secreted peptide signal through assessing induction of specific phenotypic response or lack thereof. While the predicted CSP did not induce competence, it demonstrated CSP-mediated inhibition of other streptococci, including *S. intermedius*, *S. anginosus*, *S. constellatus* and *S. vestibularis* [68].

A separate study by Aymeric and colleagues went on to investigate the potential effects of *Sgg* gut colonization as it relates to colon cancer development. Mice with a CRC-predisposing genetic mutation had greater gut colonization by *Sgg*, and this colonization was happening at the expense of nearby enterococci [70]. Further studies on the predatory nature of *Sgg* revealed the production of a class II bacteriocin, termed gallocin, that is specifically produced and secreted by *Sgg* and enhanced in bile acid-enriched environments—a classic risk factor for CRC [67].

Later research focused on the elucidation of genes involved in the regulatory control of gallocin production revealed that four genes are responsible for the regulation of gallocin, including a peptide signal, an associated TCSTS, and an additional putative regulator with unknown function [71]. The TCSTS and associated signal peptide were found to be what was previously identified as the ComABCDE competence regulon, thus the mature signal (CSP) was renamed to gallocin-stimulating peptide (GSP). SAR analysis revealed the minimum peptide scaffold needed to initiate the production of gallocin. Interestingly, in the case of *Sgg*, it was found that the first nine *N*-terminal residues of the 21-mer GSP, including the *N*-terminal aspartate, were dispensable, or could be removed without significantly affecting peptide activity. This study provided substantial groundwork in rationally designing future peptide inhibitors for *Sgg* targeting gallocin production [69]. As such, inhibitors of this pathway may help prevent *Sgg* colonization and the development of CRC.

### 7.3. Mutans group: *S. mutans*

*Streptococcus mutans* is a highly cariogenic pathogen known to cause associated bacteraemia and endocarditis. Changes in environmental conditions, like increased carbohydrate concentration, promote the colonization of *S. mutans*. This species is known to establish robust biofilms and utilizes a vast array of antimicrobial agents to eliminate neighbouring species and promote its own successful colonization [72]. Great efforts have been made to understand the signalling pathways of this species to identify potent inhibitors and reduce associated disease.

*Streptococcus mutans* utilizes both the ComABCDE and ComRS systems to regulate competence as well as other phenotypes linked to virulence [73]. While the ComABCDE circuitry does aid in *comX* regulation, direct activation of this gene relies on activation of ComRS by XIP in *S. mutans* [74]. When the concentration of XIP reaches a minimum threshold, XIP is reimported back into the cell by the oligopeptide permease, OppA, forms a complex with ComR, and goes on to upregulate *comX* transcription for the entire bacterial population. XIP also leads to the upregulation of its own propeptide signal, encoded by *comS*, establishing a positive feedback loop to amplify signal and bacterial response [73].

Earlier studies utilized transcriptome analysis to identify potential peptide targets that interacted with *comX*, thereby maintaining an influence on subsequent phenotypes that aid in pathogenesis. This strategy aided in the characterization of a novel regulatory peptide, termed *comX* regulatory peptide A (XrpA), that is unique to *S. mutans* [75]. Later, researchers found that an XrpA-deficient *S. mutans* strain showed a 6.7-fold increase in transformation efficiency compared to the wild-type. Conversely, a 6.8-fold decrease in transformation efficiency was observed in an *xrpA*-overexpressing mutant [76]. This mutant also exhibited much slower growth, with and without the addition of exogenous synthetic CSP, compared to the wild-type and *comX*-overexpressing strains of *S. mutans* [76]. These results support the notion that activation of the ComABCDE circuitry using CSP is not sufficient for *S. mutans* to efficiently coordinate proliferative phenotypes. These results also confirmed that XrpA serves as an antagonist of *comX*, and that normal expression of XrpA is required for *S. mutans* to elicit desired phenotypic response.

Kaspar and co-workers had also revealed that the *N*-terminal region of XrpA is capable of inhibiting the ComR::XIP complex from binding to conserved promoter regions, similar to p*Ceb*, upstream of *comX*. This was confirmed via *in vivo* protein cross-linking and *in vitro* fluorescent polarization assays [75]. Elucidation of this mechanism confirmed that XrpA is capable of acting as an effective negative regulator of competence in *S. mutans* [73,75]. Future modification of this regulatory peptide could aid in establishing a more potent inhibitor of the ComR::XIP complex, thereby ensuring the prevention of *comX* transcription and associated proliferative phenotypes.

**Figure 7.**
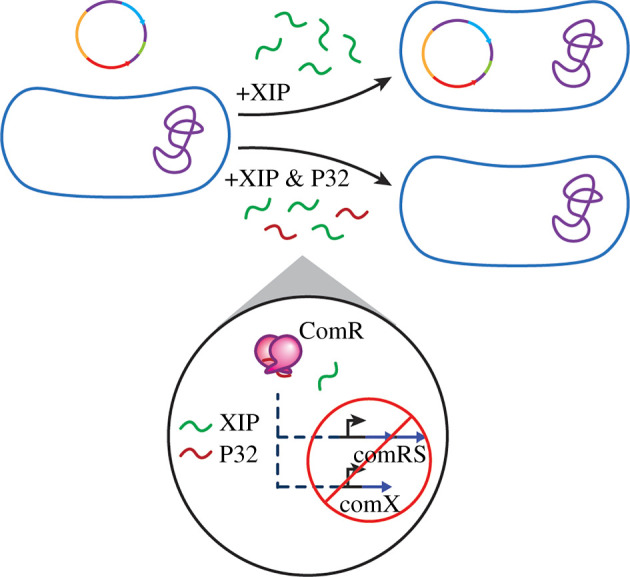
Effect of P32 in *S. mutans*. Identified inhibitor peptide, P32, prevents competence development in *S. mutans* when incubated with the bacteria in the presence of XIP.

Efforts were also directed towards further exploring the role of XIP in QS modulation in *S. mutans*. Bikash *et al*. utilized a series of peptide scans and conservative point mutations to identify residues needed for XIP receptor binding and/or activation. An alanine scan revealed that modification of the native XIP with alanine residues at positions 4 and 7 resulted in analogues capable of inhibiting ComR activation, suggesting that these residues are largely involved in receptor activation, not binding [77]. Results from the alanine scan also revealed residues that could be modified while maintaining receptor activation. Data coalesced from these scans directed the establishment of a peptide library with multiple point mutations. While multiple peptide analogues with significant inhibitory activity were found, a lead peptide (XIP-G1AD3AW4AS6A), termed P32, was identified, which had significant inhibitory activity in the presence of both the native CSP and XIP signals. These results indicate potential involvement with both the ComRS and ComABCDE pathways. Incubation with P32 prevented the uptake of spectinomycin resistance genes (figure 7) [74]. While additional experiments are needed to further elucidate methods to effectively inhibit *S. mutans* QS, these studies provide promising groundwork in the search to attenuate bacterial virulence and develop effective anti-infective therapeutics using peptides [78].

### 7.4. Group B streptococci: *S. agalactiae*

*Streptococcus agalactiae,* also known as group B Streptococcus (GBS), is β-haemolytic, opportunistic pathogen that poses a risk to infants, pregnant women, the chronically ill, and the elderly. This bacterium is commensal and can be found in the gastrointestinal and urogenital tracts of up to 40% of healthy men and women [79,80]. It can cause diseases including bacteraemia, meningitis, pneumonia, urinary tract infections, osteomyelitis and soft tissue infections [81]. GBS has been exposed to a variety of environmental niches, and as such has developed numerous mechanisms to detect changes in the external environment and make necessary adjustments to survive. GBS's ability to colonize its host and cause disease is frequently governed by one or more QS circuits. These circuits regulate bacterial behaviors like adhesion and invasion of host tissues and developing resistance to host immune responses [82,83]. Virulence factors allow GBS to multiply and spread by causing tissue damage and overcoming the host's immune system. TCSTS are frequently involved in the generation of virulence factors, and numerous TCSTS in GBS control virulence-related genes. In general, GBS has at least 22 TCSTS, but most of them are poorly defined [83]. It is still necessary to identify the peptide signals that activate TCSTS sensors, as well as define the resultant genetic and phenotypic changes associated with response regulator activation. Understanding these systems will allow researchers to develop strategies to prevent and control GBS infection. Recent elucidation of two QS circuits has helped identify potential methods to modify pathogenicity using specified AIP-like probes.

Parker and co-workers identified many GBS surface proteins, lipoproteins, and secreted proteins that modulate host binding and are controlled by a TCSTS. The GBS Rgf locus contains four genes, *rgfBDAC*, which are similar to the *S. aureus* Agr QS system. The *rgfBDAC* operon encodes an ABC transporter (RgfB), a peptide (RgfD), a sensor kinase (RgfC), and a response regulator (RgfA) [84]. RgfD has been classified as a possible QS AIP that is required for full *rgfC* expression [80]. Further investigation of this peptide sequence may help identify potential QS inhibitors with the ability to prevent phenotypic expression, virulence and pathogenicity of GBS.

Another QS-related pathway identified in GBS includes RovS, a Rgg-type transcriptional regulator, and its activator, a short hydrophobic peptide (SHP) [85]. The SHP, much like the Rgf system, is typically post-translationally processed by one or more peptidases and released extracellularly [80]. The SHP interacts directly with RovS after being imported by the Opp. This system relies on autoinduction and influences the production of fibrinogen-binding proteins and host-cell attachment [80,86]. Modification to the SHP could yield RovS inhibitors, thereby affecting the production of multiple virulence-related proteins and preventing the fruition of various pathogenic phenotypes.

### 7.5. Sanguinis group: *S*. *sanguinis* and *S. gordonii*

While the previous sections focused on the pathogenicity of selected species and methodologies to inhibit mechanisms these species use to accomplish virulence, it is also worthwhile to understand the beneficial contributions of the oral microflora. While sanguinis group streptococci are still considered opportunistic pathogens, they are also early colonizers of the oral environment and participate in commensal interactions in this community. Research has been focused on gaining a mechanistic understanding of the phenotypic responses that *Streptococcus sanguinis* and *Streptococcus gordonii* utilize to promote oral health, as well as combat invading pathogens [26,87]. As such, these phenotypes, specifically hydrogen peroxide formation, could be exploited to combat notorious oral pathogens, like *S. mutans*.

**Figure 8.**
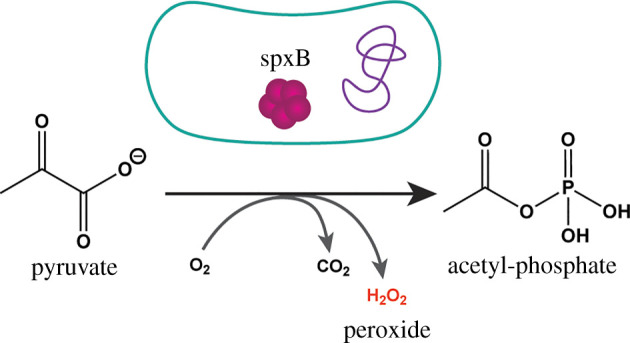
SpxB Mechanism. SpxB processes available pyruvate into acetyl-phosphate, yielding hydrogen peroxide as a side-product.

Research by Kreth and co-workers was directed toward understanding antagonistic relationships between streptococcal species, specifically those with the pathogen *S. mutans*. *S. mutans* dominance, and therefore disease, depends on competition with neighbouring species like *S. sanguinis*, as well as the presence of other hydrogen peroxide-forming species, specifically *S. gordonii*. [26] Researchers found that in an aerobic environment, both *S. gordonii* and *S. sanguinis* produce hydrogen peroxide using the previously identified pyruvate oxidase (SpxB) system in which pyruvate is processed to yield acetyl phosphate, carbon dioxide and hydrogen peroxide (figure 8) [88]. Interspecies competition assays in both aerobic and anaerobic conditions demonstrated inhibition of *S. mutans* growth due to hydrogen peroxide formation, especially in an aerobic environment. It was demonstrated that hydrogen peroxide elicited an apoptotic-like response, resulting in the expulsion of DNA materials into the extracellular space.

It was hypothesized that SpxB was in part regulated by QS, however, this was not confirmed until Moraes and co-workers [89] investigated the regulatory function of the VicRK TCSTS and its effects on *S. sanguinis* phenotypes. Researchers found that inactivation of VicK impaired the production of hydrogen peroxide in *S. sanguinis* and significantly hindered its ability in inhibiting *S. mutans*. Transcriptional analysis revealed that the VicRK system regulates genes involved in hydrogen peroxide formation, including *spxB* [89]. Modulation of the VicRK system would allow for the control of downstream genes. As such, phenotypes like hydrogen peroxide formation could be harnessed to combat *S. mutans* and prevent colonization and subsequent infection.

There has been some research devoted to investigating bacteriocin and CSP production in *S. sanguinis* and *S. gordonii*, however much remains to be discovered. While these bacteria are typically commensals with minimal pathological impact, they are still defined as opportunistic pathogens that cause disease when the opportunity presents itself. Further elucidation of specific AIP sequences and corresponding QS mechanisms will aid in further defining streptococcal disease and peptide leads that will aid in finding alternative solutions to treat infections.

## 8. Conclusion

Insight into streptococcal QS has given researchers a much more profound understanding of the mechanisms these bacteria are using to communicate and the implications of such communication. The basis of streptococcal QS lies in the mature peptide signal, which can effectively be predicted and detected. This signal can subsequently be modified to yield potent QS inhibitors or activators, as described previously. Upon activation of QS, researchers can elicit specific behaviours and phenotypes on demand, while peptide analogues with inhibitory activity can aid in preventing these same responses. The attenuation of QS with the use of peptide analogues aids in preventing the establishment of bacterial phenotypes associated with pathogenicity, including the development of antibiotic resistance. In a different light, the promotion of QS can aid in efficiently eliciting desired phenotypes to prevent pathogenicity in other streptococcal species, as was exhibited with inhibitory hydrogen peroxide formation by *S. sanguinis* to inhibit *S. mutans* growth and virulence.

While QS is highly conserved and incredibly beneficial to the overall fitness of a bacterial population, it is nonessential by nature and modification of this pathway will likely not select for a more fit strain. This is important, as this methodology circumvents the selection for antibiotic resistance among streptococci. The diminishing effectiveness of antibiotics and rapidly increasing prominence of antibiotic-resistant bacteria pushes the need for novel therapeutics to combat infections. The use of peptide-based probes to target nonessential communication pathways in streptococci shows great promise in serving as a powerful future therapeutic.

More work needs to be completed to gain a more thorough understanding of streptococcal QS. Additional SAR analyses of identified AIPs will help define potent peptide inhibitors or activators of QS and define new methods to prevent bacterial virulence and attenuate infection. Additionally, identification of new TCSTS or RRNPP circuits and their associated peptide signals will be useful in developing new methods to target QS and attenuate pathogenesis. Continual investigation of QS will also help advance current knowledge of sociomicrobiology. Combined, these will improve our understanding of effective and safe antimicrobial approaches that maintain the integrity of the natural, beneficial microflora while treating infections appropriately.

Targeting QS in streptococci will aid in reducing the selection for antibiotic-resistant species, as this method is both non-lethal and thereby non-selective when compared to traditional antibiotic treatment. As such, the use of peptide analogues has great potential to prevent the spread of antimicrobial resistance through the attenuation of QS. This will continue to eliminate or reduce excessive and inappropriate use of antibiotics, thereby preventing the continued spread of antibiotic-resistant infections. Moving away from antibiotics and exploring other modes of reliable and effective treatments will help in reducing worldwide morbidity and mortality associated with antibiotic-resistant streptococcal infections. Additionally, understanding QS to a greater extent will also aid in further delineating microbial interactions and crosstalk, yielding a more thorough understanding of the microbiome as a whole. The use of peptide-based probes to target nonessential communication pathways in streptococci shows great promise in serving as powerful therapeutic leads and will help work towards novel, peptide-based therapeutics for future clinical use.

## Data Availability

This article has no additional data.
